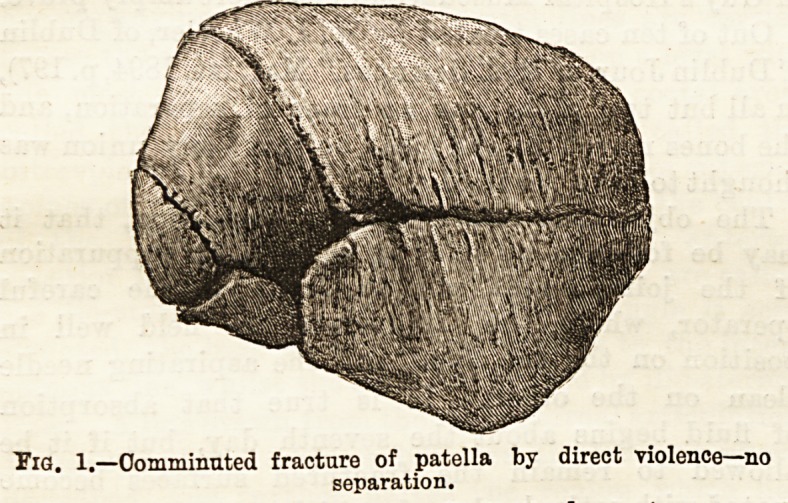# The Treatment of Fractures of the Patella

**Published:** 1896-02-15

**Authors:** John Poland

**Affiliations:** Visiting Surgeon to the Miller Hospital, Surgeon to the City Orthopædic Hospital, and Consulting Surgeon to the St. Pancras and Northern Dispensary, &c.


### Medical Progress and Hospital Clinics.

[*The Editor will be glad to receive offers of co-operation and contributions from members of the profession. All letter should be addressed to* The Editor, at the Office, 428, Strand, London, W.C.]

### THE TREATMENT OF FRACTURES OF THE PATELLA.

By John Poland, F.R.C.S., Visiting Surgeon to the Miller Hospital, Surgeon to the City Orthopædic Hospital, and Consulting Surgeon to the St. Pancras and Northern Dispensary, &c.

The subject of fractured patella is full of importance to others besides the hospital surgeon. Although the injury may not be of everyday occurrence in general practice, it is so much the more necessary to recognise the treatment of to-day in each and every variety of fracture. In this, as in other departments of surgery, the responsibilities of the practitioner become greater every day, for he must be able to instruct his patients as to the advisability of operation in any particular case. Many of the methods given in text-books have now been discarded; moreover, no single method is suitable to all cases. Considering first some of the methods of treatment which have received more general attention, allusion will then be made to the fitness of each for the various conditions met with. Two recent articles may be consulted, viz., a concise account of

Fig. 1.—Comminuted fracture of patella by direct violence—no separation.

the causation, mechanism, pathological anatomy, and treatment of patella fracture by Ferdinand Bähr, "Klin. Vorträge," N.F. No. 107 ("Chirurgie," No. 30), September, 1894, and a short comparative account by A. Carless ("Practitioner," June, 1895, p. 518) of the different methods of dealing with this fracture in the practice of surgeons belonging to the London hospitals. We may dismiss the group of comminuted, stellate, or vertical fractures from direct violence by stating that a simple retentive apparatus is all that is required, there being little or no separation of the fragments. The quadriceps extensor, periosteum, and surrounding fascia are not extensively lacerated as they are when the injury is combined with muscular action. The accompanying drawing (Fig. 1) is of a beautiful specimen of stellate fracture, caused by direct violence. It was a complication of a rare case of comminuted fracture of the head of the tibia with impaction, which was under my care, and is described in the "Transactions of the Hunterian Society," 1891, p. 116. The specimen is in the Royal College of Surgeons' Museum. There was no displacement of the fragments, and during life the fracture was not detected, the patella and joint moving freely. The following case, also under my care, shows how well these do in the ordinary way. The driver of a waggon, aged forty-four, received a kick on the left knee from a horse on Blackfriars Bridge and was able to reach the hospital on a tramcar. The usual slight separation of fragments of a stellate fracture was evident, with but little laceration of the tendon and capsule in front, although crepitus was present and a large amount of blood in the joint. The limb was immobilised on a back splint and footpiece, and later in plaster of Paris. In the course of a few weeks osseous union of the fragments had taken place without any separation, the ordinary result. A remarkable instance of transverse fracture of the patella without separation, and due to direct violence, is related by Dr. McBurney in the "Annals of Surgery," 1895, p. 312. The fracture involved the anterior surface only, a groove marking the line of injury from one side to the other, but no mobility of the patella ends upon one another. Recovery was rapid, the treatment being simply that for a mild amount of contusion of the soft parts. The man had complete power of flexion and extension, conditions quite inconsistent with separation of the fragment. The skin was not broken.

Usually the patient can get about after a fortnight with crutches, using a plaster of Paris splint for another three or four weeks.

The rare cases of fracture of the patella in children on record have mostly been vertical splits or marginal chips of the cartilaginous or incompletely ossified bone from direct blows. They should be treated like similar injuries in adults. Transverse fracture is almost unknown in young subjects.

First, as to the most simple form of treatment of transverse fractures, viz., rest in the horizontal position with the heel raised by an inclined plane or otherwise. This may be all that is necessary to effect the first great principle of treatment; that is, to bring down the upper fragment and accurately approximate the two osseous surfaces. This plan will suffice in exceptionally simple instances in which there is an absence of any great amount of blood or serum in the joint. Hamilton, in the later editions of his "Fractures and Dislocations," no longer recommended the wooden inclined plane, and Mr. Hutchinson, many years ago, stated that he considered elevation of the foot useless. I think that it is a matter of indifference whether the elevated position is maintained or not in the majority of fractures; while some consider that the contraction of the powerful quadriceps extensor is the chief cause of the separation of the fragments. The quadriceps, after division, is in a relaxed condition, and there is, therefore, no occasion for further shortening. It cannot be doubted that elevation is frequently beneficial as a supplementary aid to treatment.

After fixation of the limb and knee-joint in the extended position by a straight, well-padded back or other splint, one of the many means of bringing down the upper fragment may be employed. The pressure should be direct; the use of the fingers may do all that is requisite if only slight pressure is required. The fragments should then occupy a hole or window cut in front of a Crofts' plaster of Paris splint moulded to the lower half of the thigh and upper half of the leg. The upper fragment must always be brought into close apposition with the lower. The Middlesex Hospital plan is to apply a piece of moleskin plaster, cut out (horse-shoe shaped) at one border to fit the upper edge of the patella, extending up the front and sides of the thigh. An indiarubber accumulator is attached above to the lower ends of the front border of the moleskin, and below to the foot piece of a well-padded McIntyre or back-splint, on which the limb is placed. Numerous similar plans have been advocated by Sir Astley Cooper, Hamilton, Grant, Steavenson, and others. Callender's arrangements of weight, strapping, and pulleys are too complicated; the same may be said of the apparatus of Lonsdale, of Beach of Philadelphia, of Wyeth, and a host of others. In some of them gangrene has supervened, and in some the approximation of the fragments was by no means as effectually maintained as after the more simple plaster of Paris. Pressure upwards on the lower fragment should be carefully guarded against. This portion with the ligamentum patellæ should be evenly bandaged upwards and backwards, and kept steadily fixed in this position. No continuous upward pressure by means of any apparatus is necessary. The evil result of pressure upon the lower fragment I have seen when operating upon cases of ununited patellar fracture with separation—its fractured surface being so tilted forwards that its articular aspect was presented directly upwards, making it extremely difficult in two instances to distinguish one surface from the other.

In 1887, Dr. F. H. Alderson showed, before the West London Medico-Chirurgical Society, an interesting specimen of transverse fracture of the patella united by dense ligamentous tissue, in the centre of which was a bridge of osseous tissue. It was taken from a man whose fracture he had treated, twenty-three years before, by simple retentive apparatus, the fragments being maintained in close juxta-position. The patient had been able to use one knee as well as the other; he never walked lame, or even used a stick.

Massage has lately been employed by Dr. P. Klemm Dr. Howard Lilienthal, and a few Dutch and German surgeons in recent fractures of the patella. In Lilienthal's case ("Annals of Surgery," May, 1895) there was little separation, and by drawing upon the upper fragment it could be approximated to the lower and crepitus elicited. A posterior splint was applied. Next day massage of the entire limb was commenced, particular attention being given to the parts about the knee and the quadriceps. A figure of eight bandage was worn during the intervals of the massage, which was performed ten minutes or more twice a day, the fragments being held in apposition by an assistant. The patient was allowed to get up on the tenth day, to walk on the eleventh, still wearing the bandage. On the fortieth day she was allowed to go up stairs, massage having been continued the entire six weeks. The result was said to be perfect, the only recognisable difference being that the patella was longer than its fellow, although it was not easy to feel a furrow between the fragments. While not agreeing with this unusual treatment, one cannot help feeling that the benefit derived may be accounted for by the fact that separation of the fragments in fractures of the patella is due in great measure to effusion into the joint and not to contraction of the quadriceps. If massage were performed earlier than at present we should get a much quicker return of function in the limb.

Presuming the immediate adjustment just advocated is a matter of impossibility on account of the articular effusion of blood directly after the accident, or of synovial fluid somewhat later, such effusion should be removed by aspiration. In doing this the most careful attention to absolute cleanliness of the instruments is imperative. If blood distend the joint in simple transverse fracture the fragments should be brought at once together without waiting for absorption to take place. Separation of the fragments according to some is entirely dependent on this effusion of blood and articular fluid, and osseous union is therefore to be expected after aspiration. I may say that I have seen fractures so treated gradually stretch their uniting material and a good deal of separation ensue where the union was said by the surgeon to have been osseous. No doubt the union was very close and everything that could be desired, still it was fibrous. That osseous union does take place occasionally specimens in Guy's Hospital Museum and elsewhere amply prove.

Out of ten cases treated by W. I. Wheeler, of Dublin ("Dublin Jour. of Med. Sciences," Mar. 1st, 1894, p. 197), in all but two there was no trace of separation, and the bones moved in one piece, so that bony union was thought to have resulted.

The objections raised against aspiration, that it may be followed by refilling or even by suppuration of the joint, ought not to occur to the careful operator, where the fragments are held well in position on the one hand, and the aspirating needle clean on the other. It is true that absorption of fluid begins about the seventh day, but if it be allowed to remain the fractured surfaces become coated with articular lymph, which renders it a matter of impossibility to get as good and as close a union as when these surfaces are brought immediately together. Herein lies one of the principal differences between fracture of the patella, olecranon, &c., and other bones which do not form part of an articulation. Their fractured surfaces are bathed with synovial secretion. Formerly it was considered the correct course to approximate the fragments after the lapse of three or more days. After aspiration the fragments must be well maintained in position by one of the above-mentioned methods, or by any other of the numerous plans—Manning's bandage, Fisher's splint, Spence's plan of Malgaigne's hooks fixed in plaster, &c. The simpler the mode of fixation the better. Two pieces of stout strapping, placed one over each end of the bone and crossed behind the joint, are often quite sufficient for the purpose, in combination with some immovable splint, such as a starch bandage. Malgaigne's hooks, although given up not many years ago on account of erysipelas and suppuration, which extended to the joint, have recently been adopted again, in a modified form, by a few surgeons who carry out aseptic methods. In all the above plans the patient should be kept in bed five or six weeks, and then wear a leather, or other contrivance, for some months longer. In 1879 Mr. Macnamara re-introduced Dieffenbach's practice of subcutaneous division of the quadriceps tendon above the patella. This has, fortunately, been little employed, through the great probability of opening the joint by the extensive incision that has to be made through the broad tendon at this part. Mr. Lund brought before the Clinical Society in 1882 a method of transfixing each fragment of the bone by a steel needle passed horizontally from side to side, and holding them in apposition by a twisted suture on either side passed over the ends of the needles.

A similar plan of extra-articular suture has been described by Mr. Myles before the Royal Academy of Medicine, in Ireland. Neither of these has been extensively adopted, by reason of the danger of opening the joint at the articular surface of the patella. In Kocker's and A. E. Barker's method the suture is passed through the joint. In Barker's suture (*Brit. Med. Jour.*, Feb. 27, 1892), a well-curved needle threaded with short sterilised silk is passed vertically through the skin and joint upwards, behind the patella, and out at the skin above the bone, and again unthreaded between the skin and front of the patella. The ends of the silk are tied firmly below and cut short. The patella fragments are thus brought firmly together in a vertical direction. J. W. White, of Philadelphia, zealously supports ("Annals of Surgery," Nov., 1895, p. 661, Barker's method).

## Figures and Tables

**Fig. 1. f1:**